# Application of growth promoting hormones alters the composition and antioxidant potential of dill essential oil under salt stress

**DOI:** 10.1038/s41598-022-18717-4

**Published:** 2022-08-23

**Authors:** Kazem Ghassemi-Golezani, Neda Nikpour-Rashidabad, Samira Samea-Andabjadid

**Affiliations:** Department of Plant Eco-physiology, Faculty of Agriculture, University of Tabriz, Tabriz, Iran

**Keywords:** Biochemistry, Physiology, Plant sciences

## Abstract

The performance of dill plant may be affected by adverse environments such as salinity. Thus, this research was designed to evaluate changes in chemical composition and antioxidant activity of seed essential oil of dill (*Anethum graveolens* L.) in response to salinity (0, 5, 10 and 15 dS/m) and 1 mM of each hormonal treatments (gibberellic acid, salicylic acid, and cytokinin). Salicylic acid (SA) reduced Na^+^ content of roots and leaves by 15.4%, 30.9% and 12.4%, 24.3%, but enhanced K^+^ content by 29.8%, 51.6% and 76.6%, 73.4% under moderate and severe salinities, respectively. Essential oil yield was enhanced with progressing seed filling, despite decreasing essential oil percentage. Percentage of essential oil was increased under low and moderate salinities. Hormonal treatments, particularly SA enhanced seed mass and essential oil percentage, leading to enhanced essential oil yield. The amounts of most constituents were enhanced under moderate salinity. Foliar spray of SA and CK (cytokinin) increased almost all essential oil components, except dill ether and dill apiole, while the GA_3_ (gibberellic acid) treatment reduced most of the constituents. The α-fenchol was only induced by salt stress. The β-pinene, 1-terpineol, cryptone, oxypeucedanin hydrate, α-thujene and P-α-dimethylstyrene were also specifically synthesized in SA treated plants under salinity. The highest TPC (total phenolic content) and antioxidant activity were recorded for essential oil of SA treated plants at mass maturity under moderate salinity. In general, the SA spray was the most effective treatment for improving essential oil quantity and quality of dill plants.

## Introduction

Dill (*Anethum graveolens* L.) as an aromatic annual herbal plant of Apiaceae (Umbelliferae) family is originated from southwest Asia or southeast Europe^1^. Dill is mostly used as a flavor and spice. Furthermore, it is a natural remedy due to its medicinal properties for digestive disorders. Currently, the beneficial features of dill such as antioxidant and antimicrobial activities have been proved^2^. The analysis of dill seed demonstrates that it comprises essential oil (1–4%), fatty acids, moisture, proteins and mineral nutrients such as potassium, magnesium, calcium, phosphorous, sodium, vitamin A and niacin. The essential oil extracted from dill seed mainly contains carvone (30–60%), limonene (33%), phellandrene (20.6%), pinene, diterpene, dihydrocarvone, cineole, myrcene, paramyrcene, dill apiole, isomyristicin, myristicin, myristin, apiole and dill apiole^3^.

The accumulation of essential oils and their composition is affected by different factors, including genetic constitution^4^, harvest time and environmental conditions^5^. Chemical compositions of essential oils may vary, depending on origin and developmental stage^6^. Some studies have been laid out to investigate changes in chemical compounds and antioxidant activities of essential oil at different developmental stages of *Thymus caramanicus*^7^ and sweet fennel^8^. The adverse effects of high salinity such as toxicity, disruption in nutrient balance and subsequently a decrease in productivity can be detected at major physiological stages of plants^9^. Salt stress may also change yield and composition of essential oils in aromatic plants^10^. The impacts of salinity on essential oil production vary, depending on plant species and salt toxicity^11^. Essential oils are formed mainly by monoterpenes, which are secondary metabolites of plants. Several practices could be used to augment essential oil production and its particular compounds, among which the natural regulators has received more attention in recent years^12^.

Gibberellins (GA) are diterpenes that have been shown to affect the production and chemical composition of essential oils. Application of GA_3_ reduced the essential oil yield and led to a wide variation in the composition of essential oil in Basil (*Ocimum gratissimum* L.) plants, so that some compounds vanished and some new compounds appeared^13^. Ghassemi-Golezani and Nikpour-Rashidabad^14^ reported that pretreated dill seeds with gibberellic acid and salicylic acid enhanced essential oil production of dill plants under saline condition. Salicylic acid (SA) is an endogenous signaling molecule that plays an important role in plant defense^15^. The SA has triggering roles on secondary metabolites such as glucosinolates^16^, alkaloids^17^, and anthraquinones^18^. In addition, the SA signaling pathway is involved in the synthesis of terpenoids including sesquiterpenoids^19^, diterpenoids^20^, and triterpenoids^21^. Treatment with SA induced the activity of antioxidant enzymes and the accumulation of polyphenols and bioactive compounds in red amaranth^22^. As a plant-produced phenolic compound, the SA can stimulate phenolic compounds and the production of new poly phenols^23^. Cytokinin (CK) is another important plant hormone that stimulated the metabolism and accumulation of essential oil and also changed essential oil composition in spearmint (*Mentha spicata*)^24^ and *Thymus mastichina*^25^. However, changes in essential oil content and composition of dill seeds at different stages of development in response to salt stress and natural regulators were not documented so far. Thus, this research was focused on evaluating the effects of exogenous gibberellic acid, salicylic acid and cytokinin on essential oil accumulation, composition and antioxidant activity of dill seeds at different phonological stages under various salinities.

## Results

### Root and leaf sodium (Na^+^) and potassium (K^+^) contents

**Figure 1:**
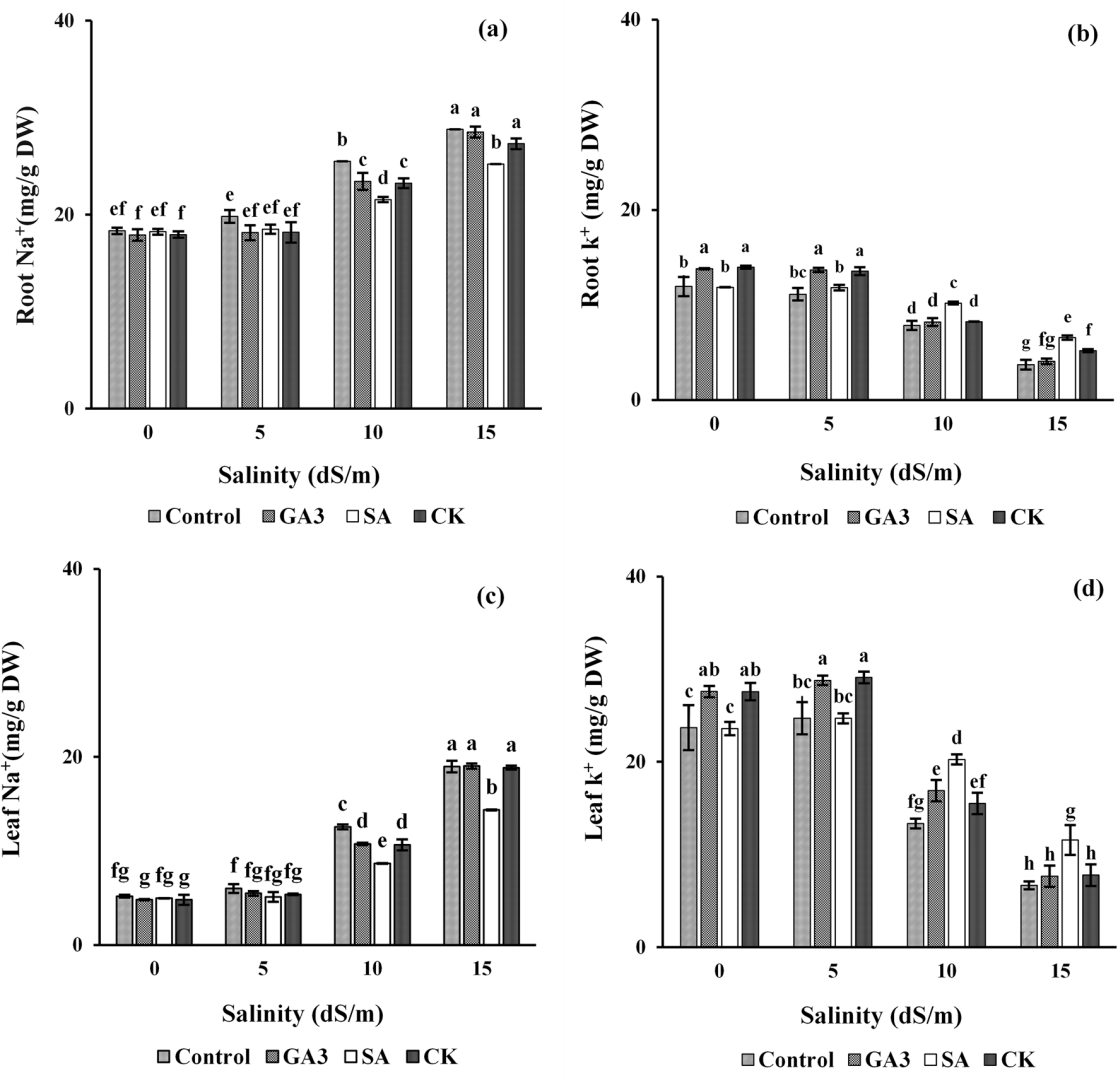
Changes in dill root and leaf sodium (**a,c**) and potassium (**b,d**) contents in response to salinity and hormonal treatments. Data represents the average of three replicates (*n* = 3) ± standard error. Different letters indicate significant differences by Duncan multiple range test at p ≤ 0.05. *GA*_*3*_*, SA, CK* gibberellic acid, salicylic acid and cytokinin, respectively.

Sodium and potassium contents of dill roots and leaves were significantly influenced by interaction of salinity × hormones (p ≤ 0.01). The differences between untreated and hormonal treated plants under non-saline and low saline conditions were not significant. The Na^+^ content of dill roots and leaves was considerably enhanced (Fig. 1a,c), while the K^+^ content was reduced (Fig. 1b,d) under moderate and severe salinities. The GA_3_, SA and CK treatments mitigated Na^+^ toxicity under moderate salinity. However, application of salicylic acid had the highest impact on reducing sodium content under moderate and severe salinities (Fig. 1a,c). In addition, exogenous GA_3_ and CK improved the K^+^ content of roots and leaves under non-saline and low saline conditions, with no significant difference between these treatments. The effect of SA on root and leaf K^+^ was more pronounced under 10 and 15 dS/m salinities (Fig. 1b,d).

### Seed filling

**Figure 2:**
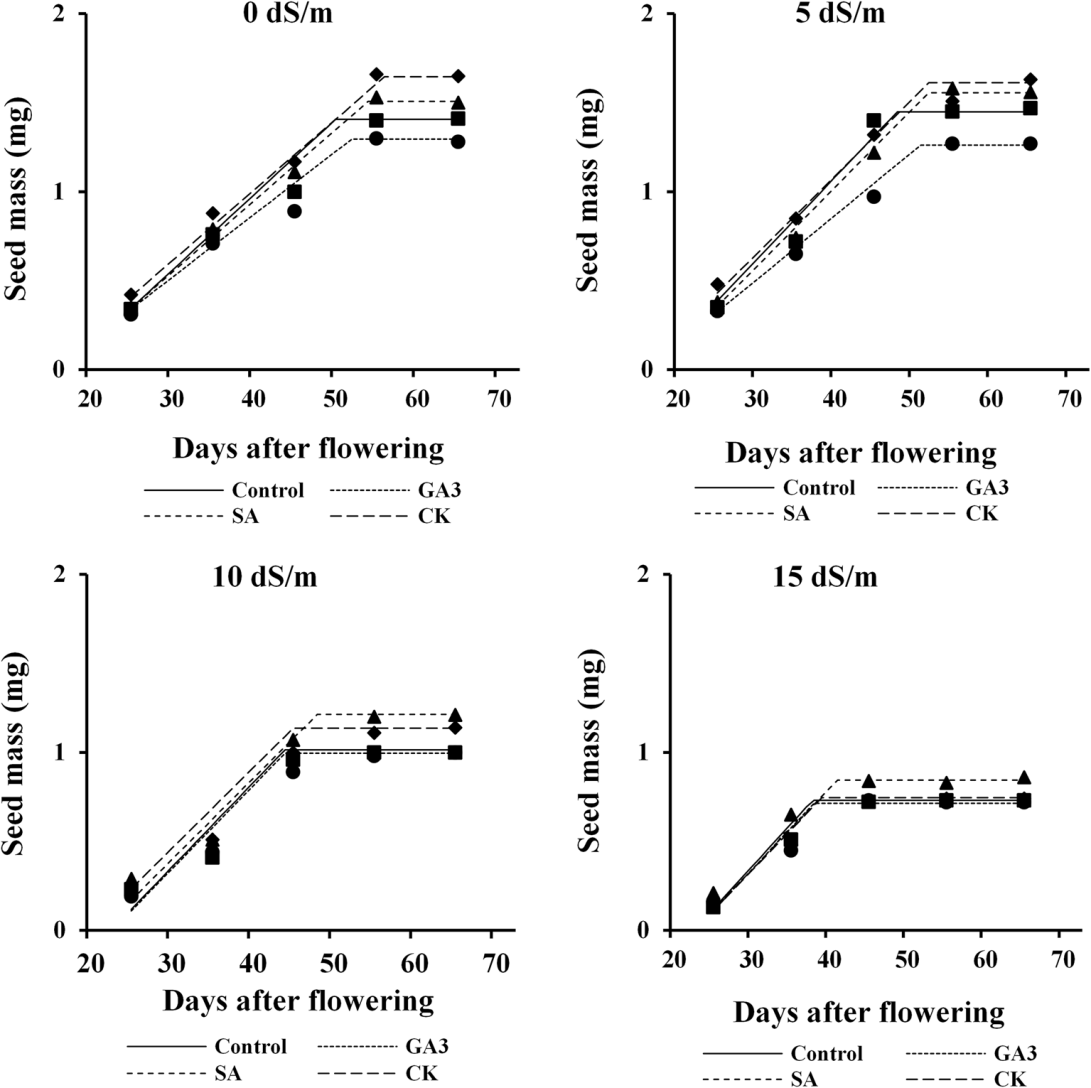
Changes in mean seed mass in response to salinity and hormonal treatments. *GA*_*3*_*, SA, CK* gibberellic acid, salicylic acid and cytokinin, respectively.

Dill seed mass was enhanced under all levels of salinity during seed development up to mass maturity (38–55 days after flowering, depending on salinity level and hormonal treatments) and thereafter no further change occurred. The highest seed mass under non-saline and low saline conditions was obtained in CK treated plants, followed by SA treated plants, while under moderate and severe salinities the largest seeds were produced by SA treated plants at later stages of development. The GA_3_ treated plants had the lowest seed mass under non-saline and low-saline conditions. Maximum seed mass for control, GA_3_, SA and CK treated plants were achieved at 50, 52, 54 and 56 days after flowering (AF) under non-salinity; at 48, 50, 52 and 53 days AF under 5 dS/m NaCl; at 44, 44, 48 and 45 days AF under 10 dS/m NaCl; and at 38, 38, 41 and 39 days AF under 15 dS/m NaCl, respectively (Fig. 2).

### Seed essential oil

**Figure 3:**
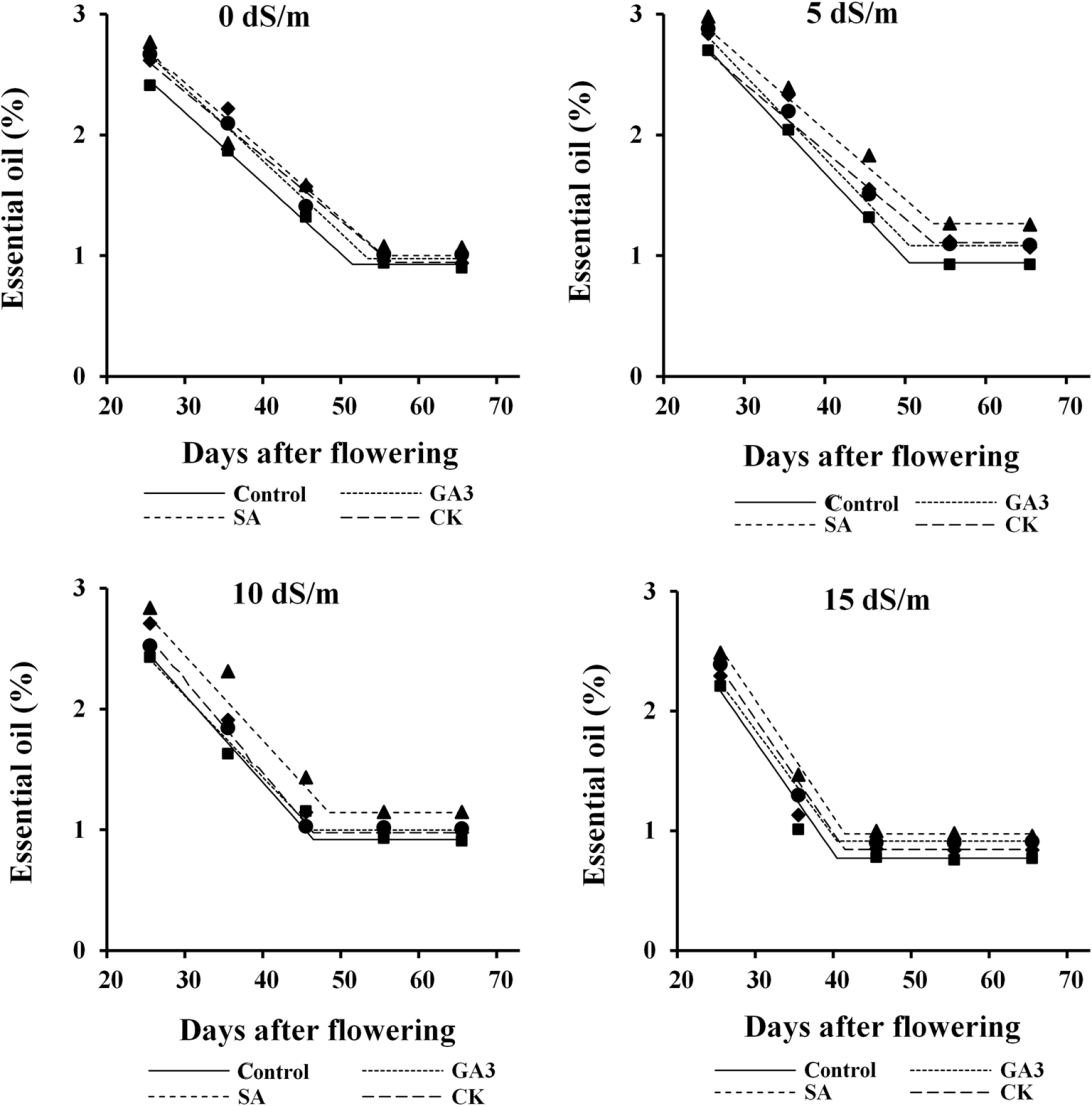
Changes in essential oil percentage during dill seed development in response to salinity and hormonal treatments. *GA*_*3*_*, SA, CK* gibberellic acid, salicylic acid and cytokinin, respectively.

**Figure 4:**
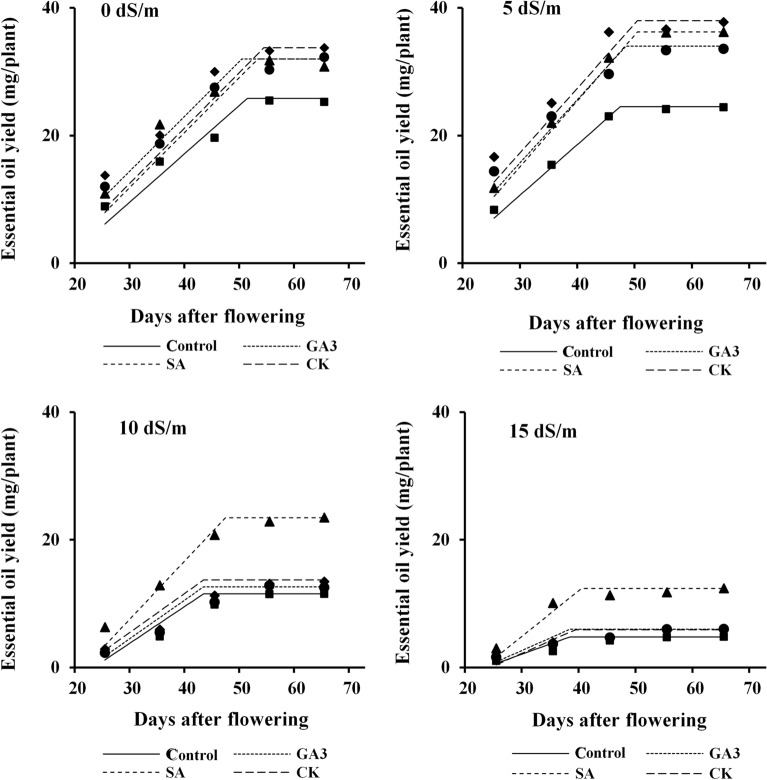
Changes in essential oil yield during dill seed development in response to salinity and hormonal treatments. *GA*_*3*_*, SA, CK* gibberellic acid, salicylic acid and cytokinin, respectively.

Essential oil percentage (Fig. 3) was gradually decreased, but essential oil yield (Fig. 4) was increased with increasing seed development up to 38–52 days after flowering, depending on salinity levels and hormonal treatments. Thereafter no changes were occurred. Essential oil percentage of dill seeds was considerably increased by low and moderate salinities, but it was significantly decreased under severe salinity. Hormonal treatments, particularly SA enhanced essential oil percentage of seeds at different developmental stages under all salinity levels. The highest essential oil percentage was obtained from immature seeds of SA-treated plants under low and moderate salinities (Fig. 3).

Maximum essential oil yield of dill seeds from untreated and hormonal treated plants in moderate and severe salinities was achieved earlier than in non-saline and low saline conditions. All hormonal treatments had a positive impact on essential oil yield under different salinity levels. However, application of CK under non-saline and low saline conditions and SA under moderate and severe salinities showed a great advantage on seed essential oil yield at all stages of seed development and maturity. The lowest essential oil yield was recorded for untreated plants under non-saline and all saline conditions (Fig. 4).

### Essential oil composition

The essential oil of dill seeds contained about 24 compounds, which were listed in Table 1. The essential oil components of hormonal treated and untreated plants were compared at 55 days after flowering under non-saline condition as control and moderate salinity as the treatment with the highest antioxidant activity of essential oil (Table 3). The percentages of most constituents were increased under moderate salinity compared to control. Treatment of dill plants with SA and CK showed positive effects on almost all essential oil components, except dill ether and dill apiole, under non-saline and moderate salinity. Application of GA_3_ reduced the amount of most constituents under both control and moderate salinity. Foliar spray of SA was the most effective treatment for increasing carvone, *N*-dihydrocarvone, iso-dihydrocarvone, α-phellandrene, β-phellandrene in dill essential oil. The β-pinene, 1-terpineol, cryptone, oxypeucedanin hydrate, α-thujene and P-α-dimethylstyrene were only synthesized in SA treated plants under salinity. The α-fenchol was specifically produced in salt stressed plants, which was largely stimulated by SA treatment. However, the paramyrcene was only produced in hormonal untreated plants under non-saline and saline conditions (Table 1).

**Table 1:** The chemical composition (%) of essential oil of dill seeds affected by hormonal treatments under non-saline and moderate salinity at mass maturity.

Compounds	0 (dS/m)				10 (dS/m)			
	Control	GA_3_	SA	CK	Control	GA_3_	SA	CK
Carvone	26.60	22.71	28.00	30.11	29.11	25.00	33.00	32.31
N-dihydrocarvone	2.71	2.20	4.82	2.81	4.20	3.92	6.51	4.62
Iso-dihydrocarvone	7.82	7.51	10.53	9.22	8.51	8.21	10.50	10.74
α-Phellandrene	1.30	1.40	3.11	2.20	1.80	1.80	3.82	2.41
β-Phellandrene	0.21	0.22	0.31	0.23	0.30	0.30	0.43	0.40
Limonene	3.42	3.21	3.80	5.00	4.12	4.23	4.31	5.20
Dill ether	0.20	0.43	0.21	0.21	0.40	0.52	0.21	0.21
Dill apiole	30.22	29.23	26.60	28.11	31.40	32.53	32.50	30.61
α-Pinene	0.01	*tr*	0.04	0.02	0.03	0.01	0.07	0.03
β-Pinene	–	–	–	–	*tr*	–	0.03	*tr*
α-Terpinene	0.11	0.09	0.18	0.15	0.16	0.11	0.25	0.15
P-cymene	0.81	*tr*	1.21	1.00	1.11	1.12	1.73	1.51
Α-Terpinolene	0.05	0.05	0.14	0.06	0.10	0.08	0.32	0.15
1-Terpineol	–	–	–	–	–	–	0.09	*tr*
Carvacrol	0.12	0.11	0.39	0.12	0.15	0.16	0.81	0.31
Myristicin	0.02	*tr*	0.04	0.04	0.05	0.08	0.20	0.11
Apiole	–	–	0.03	*tr*	0.03	–	0.04	0.02
Cryptone	–	–	–	–	*tr*	*tr*	0.02	*tr*
α Fenchol	–	–	–	–	0.08	**-**	0.25	0.13
Oxypeucedanin hydrate	–	–	–	–	*tr*	*tr*	0.06	*tr*
α-Thujene	–	–	*tr*	*tr*	–	–	0.01	–
α Fenchone	–	–	*tr*	–	–	–	*tr*	–
P-α-dimethylstyrene	–	–	–	–	–	–	0.19	*tr*
Paramyrcene	0.10	–	–	–	0.13	*tr*	–	–

*GA*_*3*_ gibberellic acid, *SA* salicylic acid, *CK* cytokinin.

### Total phenolics

There were differences in total phenol content (TPC) of essential oil at different stages of seed maturity under different levels of salinity. Total phenol was increased with seed development up to 55 days after flowering and then it was decreased at harvest maturity. The phenolics at different stages were also enhanced by salinity up to 10 dS/m. However severe salinity reduced these compounds in seed essential oil. All hormonal treatments stimulated the production of phenolic compounds at different stages under non-saline and all saline conditions. The most effective treatments were CK under non-saline condition and SA under different levels of salinity. However, the highest TPC was obtained from seed essential oil of SA treated plants under moderate salinity. The GA_3_ treatment had the lowest positive effect on producing phenolic compounds, compared with other hormonal treatments (Table 2).

**Table 2:** Total phenolic content (mg GAE/g essential oil) of the essential oil from the dill seeds under different salinity and hormonal treatments at different stages of maturity.

Salinity	Hormone	Seed formation	Immaturity	Intermediate	Mass maturity	Harvest maturity
0 (dS/m)	Control	20.97 ± 1.41^w^	24.41 ± 0.82^s–w^	28.75 ± 0.99^o–w^	31.21 ± 1.54^l–w^	29.16 ± 3.28^o–w^
	GA_3_	23.57 ± 0.78^uvw^	24.36 ± 0.59^s–w^	30.23 ± 0.39^m–w^	35.90 ± 2.79^f–r^	28.28 ± 0.36^o–w^
	SA	23.97 ± 1.07^t–w^	26.10 ± 0.89^r–w^	31.37 ± 0.31^k–w^	37.93 ± 1.57^d–o^	28.43 ± 0.29^o–w^
	CK	26.78 ± 0.6^q–w^	28.68 ± 1.43^o–w^	34.39 ± 1.85^g–u^	44.41 ± 4.08^b–g^	32.14 ± 8.93^i–v^
5 (dS/m)	Control	26.38 ± 1.77^r–w^	29.08 ± 1.73^o–w^	36.23 ± 3.7^e–r^	42.05 ± 3.17^b–k^	31.06 ± 1.69^l–w^
	GA_3_	29.30 ± 3.13^n–w^	31.56 ± 3.42^k–w^	33.57 ± 2.08^h–v^	44.70 ± 2.96^b–g^	34.84 ± 5.08^g–s^
	SA	32.63 ± 3.88^g–t^	37.33 ± 4.25^e–q^	42.85 ± 4.38^b–i^	51.33 ± 3.48^ab^	40.61 ± 4.48^c–m^
	CK	30.97 ± 3.62^l–w^	34.57 ± 3.55^g–t^	40.00 ± 4.3^c–n^	45.66 ± 3.78^b–f^	32.06 ± 1.36^j–v^
10 (dS/m)	Control	29.81 ± 0.91^m–w^	33.24 ± 1.64^h–v^	37.61 ± 2.15^d–q^	43.33 ± 2.96^b–h^	32.64 ± 4.15^h–v^
	GA_3_	32.19 ± 1.31^i–v^	35.88 ± 1.66^f–r^	40.62 ± 2.86^c–m^	46.78 ± 3.41^b–e^	37.61 ± 2.79^d–q^
	SA	36.84 ± 2.84^e–r^	42.54 ± 3.75^b–j^	49.95 ± 3.65^a–d^	56.75 ± 2.9^a^	40.07 ± 3.06^c–n^
	CK	37.83 ± 3.6^d–p^	38.48 ± 2.44^d–o^	43.58 ± 3.58^b–j^	49.67 ± 4.09^abc^	43.02 ± 4.52^b–h^
15 (dS/m)	Control	24.82 ± 0.88^s–w^	26.98 ± 1.19^p–w^	29.28 ± 1.42^m–w^	34.98 ± 2.5^g–s^	23.15 ± 2.1^vw^
	GA_3_	24.95 ± 0.57^s–w^	30.79 ± 1.67^l–w^	31.41 ± 1.73^k–w^	36.14 ± 1.95^f–r^	28.94 ± 2.88^o–w^
	SA	28.19 ± 0.88^o–w^	30.84 ± 1.52^l–w^	36.11 ± 1.93^f–r^	40.48 ± 5.32^c–m^	41.33 ± 5.45^b–l^
	CK	26.25 ± 1.18^r–w^	29.81 ± 2.1^m–w^	33.04 ± 1.55^h–v^	38.32 ± 2.29^d–o^	28.27 ± 2.3^o–w^

The values are the means of three replicates ± standard error.

*GA*_*3*_ gibberellic acid, *SA* salicylic acid, *CK* cytokinin.

Different letters in each column indicate significant difference by Duncan multiple range test at p ≤ 0.05.

### Antioxidant activity

The free radical scavenging activity of seed essential oil by the DPPH method was expressed as IC_50_ (half maximal inhibitory concentration). The lower IC_50_ value indicates a strong ability of the dill essential oil for radical scavenging. Antioxidant activity of essential oil in untreated and hormonal treated plants under non-saline and all saline conditions gradually increased with seed development up to mass maturity and then slightly decreased. The highest ability of dill essential oil for radical scavenging was obtained in untreated and hormonal treated plants under moderate salinity (10 dS/m). Nevertheless, foliar spray of SA was the superior treatment for improving the antioxidant capacity of dill essential oil (Table 3).

**Table 3:** Antioxidant activity (IC_50_ = mg/mL) of the essential oil from the dill seeds under different salinity and hormonal treatments at different stages of maturity.

Salinity	Hormone	Seed formation	Immaturity	Intermediate	Mass maturity	Harvest maturity
0 (dS/m)	Control	16.09 ± 0.79^a^	15.33 ± 0.66^ab^	15.05 ± 0.96^abc^	9.82 ± 0.28^c–r^	10.11 ± 0.73^b–q^
	GA_3_	14.85 ± 1.4^a–d^	14.36 ± 0.94^a–e^	12.83 ± 1.2^a–i^	8.83 ± 0.33^f–s^	10.50 ± 0.4^b–p^
	SA	13.41 ± 0.7^a–g^	12.90 ± 0.72^a–h^	11.87 ± 0.35^a–j^	7.61 ± 0.69^h–s^	8.64 ± 0.24^f–s^
	CK	11.92 ± 0.21^a–j^	11.20 ± 0.23^a–n^	9.10 ± 0.51^e–r^	7.05 ± 0.12^j–s^	7.28 ± 0.4^j–s^
5 (dS/m)	Control	11.67 ± 0.88^a–k^	10.81 ± 0.76^a–o^	10.07 ± 0.03^b–q^	8.80 ± 0.66^f–s^	10.03 ± 0.26^b–q^
	GA_3_	11.52 ± 0.76^a–k^	9.99 ± 0.006^b–q^	9.20 ± 0.2^e–r^	7.13 ± 0.36^j–s^	8.66 ± 0.33^f–s^
	SA	9.40 ± 0.4^e–r^	8.43 ± 0.33^g–s^	7.33 ± 0.16^j–s^	5.39 ± 0.29^o–s^	6.63 ± 0.43^j–s^
	CK	10.92 ± 0.47^a–n^	9.33 ± 0.33^e–r^	8.53 ± 0.29^g–s^	6.56 ± 0.28^j–s^	8.10 ± 0.47^g–s^
10 (dS/m)	Control	11.20 ± 0.57^a–n^	10.11 ± 0.46^b–q^	8.50 ± 0.29^g–s^	5.84 ± 0.48^m–s^	9.60 ± 0.23^d–r^
	GA_3_	9.32 ± 0.39^e–r^	8.53 ± 0.31^g–s^	7.36 ± 0.55^i–s^	5.32 ± 0.18^p–s^	6.66 ± 0.33^j–s^
	SA	6.88 ± 0.52^j–s^	5.93 ± 0.54^l–s^	4.82 ± 0.1^qrs^	3.66 ± 0.32^s^	5.06 ± 0.52^p–s^
	CK	8.60 ± 0.2^g–s^	7.40 ± 0.63^i–s^	6.20 ± 0.11^k–s^	4.40 ± 0.21^rs^	5.75 ± 0.14^n–s^
15 (dS/m)	Control	14.07 ± 0.26^a–f^	11.93 ± 0.74^a–j^	10.33 ± 0.34^b–p^	8.78 ± 0.4^f–s^	10.87 ± 0.41^a–o^
	GA_3_	13.57 ± 0.69^a–g^	11.33 ± 0.17^a–m^	9.71 ± 0.35^c–r^	8.90 ± 0.46^f–s^	10.53 ± 0.84^b–p^
	SA	11.40 ± 0.58^a–l^	9.52 ± 0.2^d–r^	7.73 ± 0.14^h–s^	6.33 ± 0.24^k–s^	8.38 ± 0.61^g–s^
	CK	13.50 ± 0.29^a–g^	10.50 ± 0.5^b–p^	9.43 ± 0.35^e–r^	9.16 ± 0.19^e–r^	10.20 ± 0.25^b–q^

The values are the means of three replicates ± standard error.

*GA*_*3*_ gibberellic acid, *SA* salicylic acid, *CK* cytokinin.

Different letters in each column indicate significant difference by Duncan multiple range test at p ≤ 0.05.

## Discussion

The Na^+^ is a toxic ion which is quickly absorbed by root cells^26^. Thus, disruptions in the Na^+^ exclusion process at the root level and subsequently its translocation to the shoot under salt stress were led to a significant enhancement of this ion in roots and leaves of dill plants (Fig. 1a,c). High level of Na^+^ interferes with K^+^ uptake via common transport systems^27^, thereby reducing this cation in plant roots and leaves (Fig. 1b,d). The K^+^ has a key role in enzymes activation, membrane polarization, cell turgor, stomata movement and osmotic adjustment^28^. Hence, salt stress results in physiological disorders through imposing Na^+^ toxicity and ion imbalance^15^. However, one of the most beneficial strategies to ion balance in cytoplasm is reducing Na^+^ uptake and limiting its accumulation in plant tissues and eventually increasing K^+^/Na^+^ ratio^29^. Mitigating salt toxicity by growth regulators, especially by SA (Fig. 1a,c) may be related with increasing Na^+^ accumulation in vacuoles by activating H^+^-pumps, which reduces the Na^+^ in cytosol^30^. Foliar spray of CK also regulates fundamental cellular pathways and preserves them from Na^+^ toxicity with an increment in K^+^ uptake and K^+^/Na^+^ ratio^31^.

Reduction of maximum seed weight (Fig. 2) due to salinity could be attributed to deleterious effects of high Na^+^ content (Fig. 1a,c) on water availability and shorter filling duration, leading to early plant senesces^32^. These results are strongly supported by Ghassemi-Golezani and Nikpour-Rashidabad^14^ in dill and Ghassemi-Golezani et al.^33^ in soybean. PGRs are specifically involved in regulating sink strength, photosynthate partitioning and phloem unloading^34^. Treatment with CK increased maximum seed mass under non-saline and low saline conditions (Fig. 2). Seeds are powerful carbohydrate sinks during development^35^, and cytokinin regulates the sink size of the seed through prolonging seed filling duration. Foliar spray of SA increased assimilate mobilization by decreasing Na^+^ and increasing K^+^ contents (Fig. 1), leading to longer filling duration and larger seed production under moderate and severe salinities.

Higher essential oil percentage at early stages of development (Fig. 3) might be related to the pattern of reserves accumulation in seeds. In fact, essential oil in plants of Apiaceae family accumulate in some tubes called “vittae’ that are abundant in immature seeds. Reduction of essential oil in mature seeds might be caused by further accumulation of other photosynthates to the seeds at later stages of development^36^. Enhancing essential oil production under low and moderate salinities and reducing that under severe salinity (Fig. 3) could be attributed to adverse impact of high Na^+^ content (Fig. 1a,c) on biosynthesis of primary and secondary metabolites^37^. Foliar spray of SA remarkably improved essential oil content by mitigating the adverse effects of salt stress on plant growth and metabolism through reducing Na^+^ uptake (Fig. 1a) and accumulation in leaves (Fig. 1c).

Despite variation in essential oil percentage (Fig. 3), the increment of essential oil yield was largely related with seed mass (Fig. 2). The levels of the active substances of plant metabolism change from juvenile to mature phases and they are also affected by environmental conditions. Increasing essential oil yield under low salinity (Fig. 4) can be due to maximum essential oil percentage stimulated by this level of salinity (Fig. 3). Environmental stresses may induce essential oil accumulation, as antioxidant compounds, to alleviate oxidative stress caused by salinity^38^. Reduction of essential oil yield of dill seeds with further increment of salinity (Fig. 4) may be related to large reduction in seed yield per plant. The endogenous level as well as the exogenous application of PGRs can influence essential oil production through their effect on plant growth and metabolism^39^. Foliar spray of SA was the superior treatment for enhancing essential oil yield under moderate and severe salinities (Fig. 4), which might be achieved by reducing Na^+^ and increasing K^+^ content (Fig. 1) and essential oil percentage (Fig. 3). High essential oil percentage (Fig. 3) of CK treated plants was also resulted in higher essential oil yield under non-saline and low saline conditions (Fig. 4).

The major oil constituents of dill essential oil are terpenes (carvone, phellandrene and limonene), which are synthesized through two pathways of mevalonate and methylerythritol phosphate. The biosynthesis and accumulation of these compounds are mostly related to internal factors such as developmental stage of whole plant and specific organ and external factors such as environmental stresses. Improving the content of each constituent in dill essential oil under moderate salinity (Table 1) could be attributed to the effect of this stress on synthesis of some secondary metabolites^40^ and the function of these metabolites as self-defense components against stressful conditions. Furthermore, salinity may modify essential oil biosynthesis through marked changes in biochemical and physiological processes^41^. Thus, cultivating dill plants under moderate NaCl salinity can induce production of volatile substances. The SA and CK promoted the production of essential oil compounds under non-saline and saline conditions. However, the superiority of SA was more pronounced under moderate salinity (Table 1). It seems that these plant growth regulators stimulated terpene biosynthesis through up-regulating the terpenoid biosynthetic pathway at the transcriptional level^42^. Furthermore, this could be achieved through direct effects of these hormones on metabolism and enzymes activities responsible for mono or sesquiterpene-biosynthesis^43^. Enhancing essential oil constituents by SA treatment under saline condition could be also related to decreasing Na^+^ and increasing K^+^ contents in dill plants (Fig. 1). Decrement of some monoterpenes of essential oil and increment of dill ether and dill apiole by GA_3_ treatment (Table 1) may be associated with the impact of this hormone on effective genes activities and synthesis pathways. These effects were also reflected in induction of producing some important constituents specifically by salinity or SA treatments (Table 1).

Environmental stresses such as salinity can cause oxidative stress^44^. The production of secondary metabolites (terpenoids and phenolic compounds) is a mechanism to counter such stresses^45^ through participating in plant protection against reactive oxygen species (ROS). Phenolic compounds are synthesized when aerobic respiration or photosynthetic metabolisms are disrupted by environmental constraints^46^. Thus, considerable increment in TPC of dill seeds under salinity (Table 2) might be the result of plant resistance mechanisms to adapt the adverse environmental conditions^47^. Enhancement of TPC in dill seeds with application of natural regulators (Table 2) might be due to an increment in K^+^ content (Fig. 1b,d). Potassium has an important role in activation of most enzymes in biosynthesis pathways^48^. Furthermore, SA might induce the production of plant defensive metabolites and antioxidant enzymes^49,50^. Hence, effect of SA on TPC of dill seeds under saline conditions (Table 2) could be related to its important signaling role against salt stress^51^. The SA contributes in controlling defense-related genes expression^52^ and induces the expression of genes responsible for enzymes involved in production of polyphenolics^53^ such as PAL (phenylalanine ammonia lyase) both at the transcriptional and translational levels^54^. The CKs influence the shikimate/phenylpropanoid pathways in the biochemical synthesis of phenolic compounds possibly by regulating the activity of PAL and chalcone synthase^55^. The impacts of CKs on occurrence, distribution and contents of phenolic compounds confirm the importance and long-lasting role of CKs in phenolic biosynthesis pathways^56^. The increment of phenolic acids contents in GA_3_ treated plants (Table 2) can be attributed to induction of phenylalanine ammonia lyase and tyrosine amino transferase activities^57^.

It seems that enhancing the antioxidant activity of seed essential oil at various developmental stages by hormonal treatments, especially SA, under different salinity levels (Table 3) was related to increasing phenolics (Table 2) and some of the essential oil components (Table 1). Mass maturity was the best stage for harvest to produce high essential oil enriched with phenolic compounds (Table 2), carvone, *N*-dihydrocarvone and Iso-dihydrocarvone (Table 1). Promotion of antioxidant activity in SA and CK treated plants was achieved by increasing phenolic compounds and oxygenated monoterpenes. However, the high antioxidant activity in GA_3_ treated plants was related to production of phenolic compounds, since this hormone reduced the synthesis of most monoterpene compounds (Table 1). Phenolic compounds with high redox potential act as radical scavengers, metal chelators, hydrogen donors, reducing agents and singlet oxygen quenchers^58^. On the other hand, monoterpenes like carvone also was characterized with potent DPPH radical scavenging activity^59^. Carvone has the most powerful effect on the superoxide anion scavenging activity^60^. Therefore, dill seed essential oil with high carvone (Table 1) and phenolics (Table 2) contents is a potential source of natural antioxidant agents, which can be improved by hormonal treatments, particularly by SA spray.

## Conclusion

With progressing seed development, essential oil yield increased, despite decreasing essential oil percentage. Although salinity up to 10 dS/m caused an increase in essential oil percentage of dill seeds, essential oil yield decreased. Hormonal treatments, particularly SA enhanced essential oil percentage of seeds, leading to enhanced essential oil yield. The amounts of most constituents were enhanced under moderate salinity. Application of SA and CK increased almost all essential oil components, except dill ether and dill apiole, while the GA_3_ treatment reduced most of the constituents. The α-fenchol was only induced by salt stress. The β-pinene, 1-terpineol, cryptone, oxypeucedanin hydrate, α-thujene and P-α-dimethylstyrene were also specifically produced in SA treated plants under salinity. The highest TPC and antioxidant activity were recorded for essential oil of SA treated plants at mass maturity under moderate salinity. In general, the SA spray was the superior treatment for improving salt tolerance, essential oil quantity and quality, total phenolics and antioxidant activities of dill plants.

## Materials and methods

### Experimental design

The experiment was undertaken in a greenhouse at the University of Tabriz, with a factorial arrangement on the basis of randomized complete block design with three replications, to investigate changes in seed essential oil content and composition and also antioxidant activity of this secondary metabolite in dill (Isfahan ecotype). This dill ecotype can grow up to 40–60 cm, with hollow stems and soft leaves. The leaf divisions are 1–2 mm broad, with harder texture. The flowers are white, in umbels with 2–9 cm diameter. The seeds are usually 4–5 mm long and 1 mm thick. Treatments were non-saline and different saline conditions (5, 10 and 15 dS/m as low, moderate and severe salinities, respectively) and foliar application of water (control), gibberellic acid (GA_3_, 1 mM) salicylic acid (SA, 1 mM) and cytokinin (CK, 1 mM). The concentrations of PGRs were chosen on the bases of our previous work on this plant^14^. The day and night mean temperatures in the greenhouse were 28 °C and 26 °C, respectively. Each of 52 plastic pots (48 sown and 4 unsown) were filled with 900 g perlite. Dill seeds were pretreated with 2 g/kg Benomyl, and then 30 seeds were sown in 1.5 cm depth of each pot. Subsequently, tap water (EC = 0.59 dS/m) and specific saline solutions were added to the pots to achieve 100% field capacity (FC). After seedling establishment, plants in each pot were thinned to keep 15 plants per pot. During the experiment, unsown pots were weighed regularly and the losses were compensated by Hoagland solution (EC = 1.3 dS/m, pH 6.5–7.0). The perlites within the pots were washed every 20 days to avoid excess salinity due to adding Hoagland solution. After that, salinity treatments were reapplied. The hormones were sprayed on plants at vegetative and reproductive stages.

### Sodium and potassium contents

Two plants were removed from each pot at seed formation stage and roots and leaves were cut and dried at 80 °C for 48 h. Thereafter, 1 g of dry root and 1 g of dry leaf were separately powdered and burned at 560 °C, and the ashes were digested in 10 mL of 1 N HCl. The Na^+^ and K^+^ contents of each root or leaf samples were then measured by a flame photometer (Corning flame photometer, 410).

### Mean seed weight

Three dill plants from each pot were harvested in each of five stages (25, 35, 45, 55, and 65 days after flowering). Seeds were separated and air-dried at room temperature (18–25 °C). Then, mean dry weight of seeds was calculated for every sample. A two-pieces linear regression model was applied to estimate changes in seed dry weight at different developmental stages^61^.

$${\text{W}} = \left\{ {\begin{array}{*{20}l} {{\text{a }} + {\text{ bt}}} \hfill & {{\text{t }} < {\text{ t}}_{{\text{m}}} } \hfill \\ {{\text{a }} + {\text{ bt}}_{{\text{m}}} } \hfill & {{\text{t }} \ge {\text{ t}}_{{\text{m}}} } \hfill \\ \end{array} } \right.,$$

where W is dry weight; a is intercept; b is slope indicating seed filling rate; t is days after flowering and tm is time of mass maturity (end of seed filling).

### Extraction of essential oil

Essential oils were extracted from air-dried seeds of each treatment by water-distillation using a Micro-clevenger for 3 h. The resulted distillate was extracted using diethyl–ether as solvent (1/1, v/v), which dehydrated over anhydrous sodium sulfate. Finally, essential oil samples were kept in dark glass bottles at 4 °C until GC–MS analysis. Essential oil percentage and yield were calculated by the following formulas:

$${\text{Essential oil percentage }} = \, \left[ {{\text{essential oil }}\left( {\text{g}} \right)/{\text{seed dry weight }}\left( {\text{g}} \right)} \right] \, \times { 1}00,$$

$${\text{Essential oil yield }} = {\text{ essential oil percentage }} \times {\text{ seed dry weight}}.$$

### GC/MS analysis

Oil components of dill seeds were analyzed by gas chromatography-weight spectrometry (GC–MS) instrument. GC/MS analysis was performed on an Agilent Technologies 7890 gas chromatograph coupled to Agilent 5975 C weight spectrometer and quadruple EI weight analyzer (Agilent Technologies, Palo Alto, CA, USA). The HP-5 capillary column with 30 m length, 0.25 mm inner diameter and 0.25 µm film thicknesses was used. Split injection was conducted with a split ratio of 100:1, and one microliter of each sample was injected to the GC/MS analyzer. The temperature was programmed from 40 °C (for 4 min) to 250 °C (for 5 min) at 6 °C/min ramp rate. The carrier gas was helium at a flow rate of 1.2 mL/min. Weight range was from 40 to 400 m/z. The temperature of injector, electron impact ionization (EI) interface and ion source was 250 °C, 250 °C and 200 °C, respectively. The identification of components was based on a comparison of their GC retention indices and weight spectra with those reported in the Wiley and NIST (National Institute of Standards and Technology).

### Total phenolic content

Total phenolic content of the essential oil was determined by the Folin–Ciocalteu reagent described by Lowman and Box^62^. Gallic acid was used as a standard. The essential oil was diluted to a suitable concentration for analysis. A 0.5 mL of the essential oil, 1 mL Folin–Ciocalteu reagent and 1 mL of 2% (w/v) of Na_2_CO_3_ were mixed. After 2 h at ambient temperature, the mixture was centrifuged for 10 min (8000 rpm) and absorbance of each mixture was measured at 760 nm. A standard curve was obtained using 10–100 µg/mL gallic acid. Total phenolic content was recorded as mg gallic acid equivalents per gram of the essential oil.

### Antioxidant activity

Antioxidant assay was carried out by scavenging of the stable 2,2-diphenyl-1-picrylhydrazyl (DPPH) radical according to Wang et al.^63^. Different concentrations of essential oil (250, 500, 750, and 1000 µg/mL) were added to ethanol solution of DPPH radical (0.1 mM). The mixtures were kept at room temperature in dark condition for 30 min. The absorbance of solution was measured at 517 nm by a spectrophotometer (Dynamica, Halo DB-20-UV–Visible Spectrophotometer, United Kingdom). DPPH solution absorbance was decreased with increasing DPPH radical scavenging activity. The antiradical activity was calculated using the following equation and expressed as IC_50_:

$${\text{DPPH radical scavenging }}\left( \% \right) \, = \, \left( {{\text{A}}_{{{\text{blank}}}} {-}{\text{ A}}_{{{\text{sample}}}} /{\text{A}}_{{{\text{blank}}}} } \right) \, \times { 1}00.$$

DPPH solution and BHT (Butylated hydroxytoluene) were used as blank and positive control, respectively.

### Statistical analysis

Analysis of variance appropriate to the experimental design was conducted, using SPSS 16 and SAS 9.1.3 software. Means of each trait were compared according to Duncan multiple range test at p ≤ 0.05.

### Statement of compliance

This experimental research on plants complies with relevant institutional, national, and international guidelines and legislation.

## Data Availability

The necessary information is available from the corresponding author on reasonable request.
